# Circadian rhythms in the Drosophila eye may regulate adaptation of vision to light intensity

**DOI:** 10.3389/fnins.2024.1401721

**Published:** 2024-05-30

**Authors:** Richard Brent Nolan, Jin-Yuan Fan, Jeffrey L. Price

**Affiliations:** Division of Biological and Biomedical Systems, School of Science and Engineering, University of Missouri – Kansas City, Kansas City, MO, United States

**Keywords:** bride of double time, TRPL, Gα, rhodopsin, cry

## Abstract

The sensitivity of the eye at night would lead to complete saturation of the eye during the day. Therefore, the sensitivity of the eye must be down-regulated during the day to maintain visual acuity. In the Drosophila eye, the opening of TRP and TRPL channels leads to an influx of Ca^++^ that triggers down-regulation of further responses to light, including the movement of the TRPL channel and Gα proteins out of signaling complexes found in actin-mediated microvillar extensions of the photoreceptor cells (the rhabdomere). The eye also exhibits a light entrained-circadian rhythm, and we have recently observed that one component of this rhythm (BDBT) becomes undetectable by antibodies after exposure to light even though immunoblot analyses still detect it in the eye. BDBT is necessary for normal circadian rhythms, and in several circadian and visual mutants this eye-specific oscillation of detection is lost. Many phototransduction signaling proteins (e.g., Rhodopsin, TRP channels and Gα) also become undetectable shortly after light exposure, most likely due to a light-induced compaction of the rhabdomeric microvilli. The circadian protein BDBT might be involved in light-induced changes in the rhabdomere, and if so this could indicate that circadian clocks contribute to the daily adaptations of the eye to light. Likewise, circadian oscillations of clock proteins are observed in photoreceptors of the mammalian eye and produce a circadian oscillation in the ERG. Disruption of circadian rhythms in the eyes of mammals causes neurodegeneration in the eye, demonstrating the importance of the rhythms for normal eye function.

## Introduction

The Drosophila eye is the principal organ in the adult fly in which visual signals are initiated, and it is also an important source of circadian rhythms. Visual signals are generated by interactions of light with multiple Rhodopsins that are expressed in eight different photoreceptors cells, which become depolarized in response to light to trigger the release of neurotransmitters that engage different neural pathways in the brain, allowing for pattern recognition and color perception (reviewed in Hardie and Juusola, 2015). In addition, the eye expresses a circadian rhythm. As in other tissues, the oscillation of Period (PER) and Timeless (TIM) proteins (the latter targeted by the cryptochrome CRY) generate a circadian cycle. CRY determines the phase of the circadian cycle in the eye and not the Rhodopsins, although visual signals from the eye can control the phase of the oscillation of central brain neurons that are synaptic targets of the photoreceptors (reviewed in Helfrich-Forster, 2020). The central brain circadian neurons include approximately 150 neurons within the brain, including eight small lateral neurons that secrete that neuropeptide PDF. These PDF neurons control circadian locomotor activity via interactions with other circadian neurons in the brain and respond to multiple input pathways by which light entrains (or sets the phase of) the rhythm. These pathways include the intracellular circadian CRY receptor, expressed within the brain neurons as well as the eyes, as well as the photoreceptors of the compound eye and the Hofbauer–Buchner (HB) eyelet (Helfrich-Forster, 2020).

What is the function of the intracellular circadian pathway in the Drosophila compound eye? The fly eye is known to undergo dramatic changes in visual sensitivity that allow it to function in both low light conditions and high light conditions (Song and Juusola, 2017). The daily light:dark cycle produces the dramatic changes in light intensity to which vision must adapt, and it also produces the phase of the circadian clock. Might the circadian clock contribute to the timing of visual sensitivity in the fly eye? The fruit fly *Drosophila melanogaster* is an exceptionally well developed system for the genetic analysis of both circadian rhythms and vision, and it offers the opportunity to address this question in great depth.

## The visual transduction pathway in the Drosophila eye

Photons of light trigger the rearrangement of retinal in the eye rhodopsin from the cis to the trans conformation, and this in turn triggers a conformational change in the major Rhodopsin 1 (NinaE) that allows it to interact with and activate a Gqα protein. Gqα binds GTP, is released from the β-γ subunits, and then activates the major phospholipase C (PLC; NorpA) in the eye. This PLC cleaves phosphatidyl-inositol 4,5 bisphosphate (PIP_2_) in the photoreceptor cell membrane to generate inositol 1,4,5 trisphosphate (InsP_3_), diacylglycerol and protons. Somehow this signal is recognized by two cation channels (TRP and TPRL) that open to allow Na^+^ and Ca^++^ into the cell, thereby depolarizing the photoreceptor to generate the neural signal for light (reviewed in Hardie and Juusola, 2015; Montell, 2021).

Studies that have investigated whether any of these three PLC products or their fatty acid break down products directly trigger the opening of the TRP and TRPL channels have not produced clear evidence that any of the three are direct effectors of channel opening (Hardie and Juusola, 2015; Montell, 2021). Another possibility is that the PLC activity changes the properties of photoreceptor membrane protrusions called rhabdomeres by eliminating PIP_2_. The rhabdomeres consist of multiple 50 nm thick membrane protrusions that are produced by actin filaments, and they contain the light sensing rhodopsin molecules in the eye. It is possible to measure changes in the thickness of the rhabdomeres produced by PLC activity within seconds of light exposure via atomic force microscopy (Hardie and Franze, 2012). The change in the rhabdomeres may then produce the opening of the TRP and TRPL channels; these channels are similar to touch sensitive channels and may respond to the change in membrane conformation rather than directly to the products of PLC activity (Hardie and Franze, 2012).

**Figure 1:**
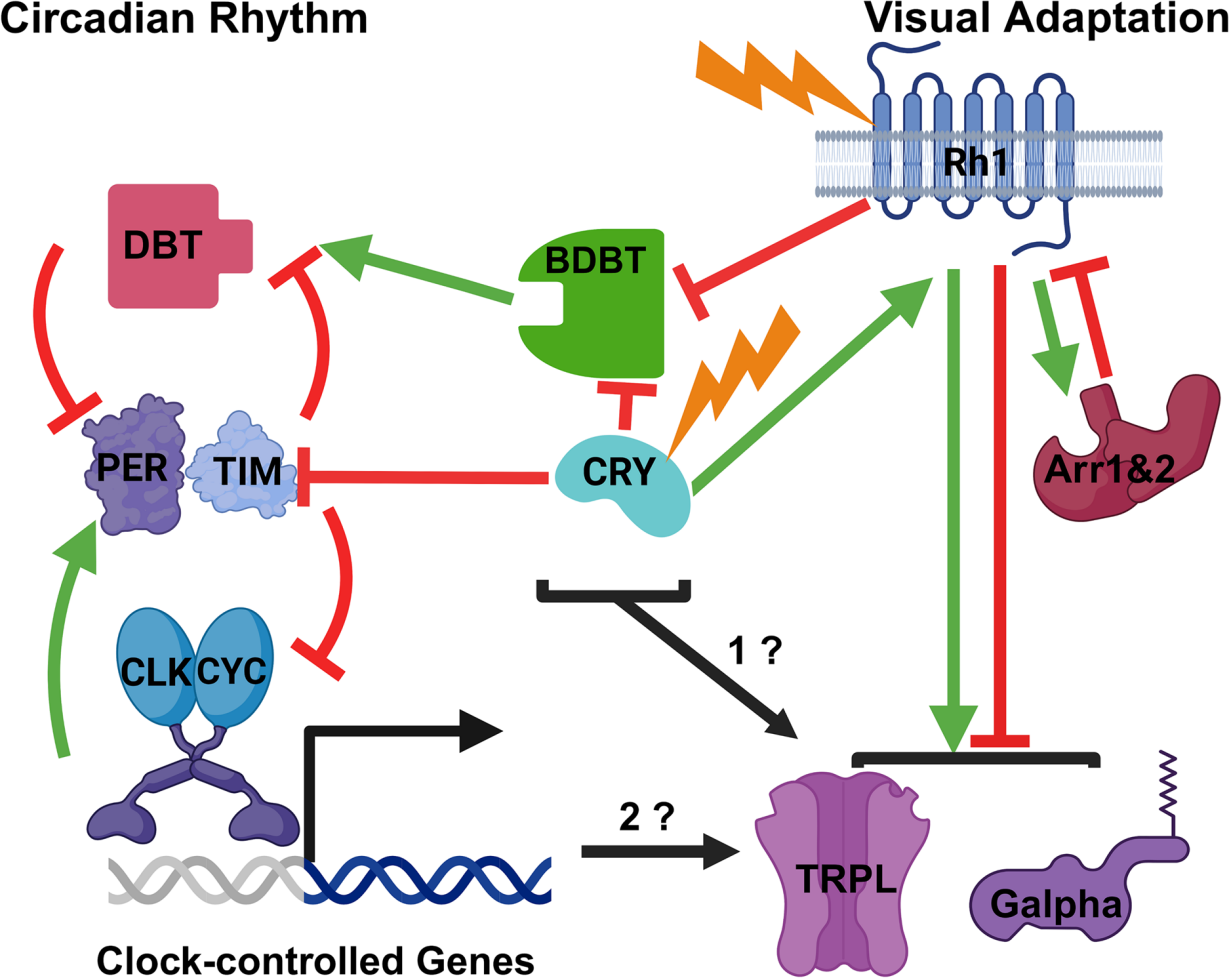
Possible Modes of Interaction between the Drosophila Circadian Clock Mechanism and Visual Adaptation to Light. A broad outline of the circadian mechanism is on the left and of visual adaptation to light on the right. Stimulation is indicated by green arrows and inhibition by red inhibition lines. The proteins and light inputs are described in the text. Based on the known effects of light and circadian rhythms on BDBT foci in the eye, BDBT might be a common element to both pathways. Likewise, the CRY photoreceptor is known to be involved in both visual and circadian pathways, and both BDBT and CRY may regulate the visual adaptation pathway (1?). Finally, it is also possible that the transcriptional oscillations produced by the circadian clock control the timing of Gα and TRPL movements for visual adaptation (2?). Created with BioRender.com.

In addition to the positive effects of increased Ca^++^, the increased Ca^++^ leads to negative regulation of the light response via effects on the TRP/TRPL channels to inactivate them, activation of protein kinase C and consequent inactivation of PLC and TRP, and interactions with a myosin III (NinaC), which is triggered to release Arrestin 2, thereby producing negative regulation of Rhodopsin 1 (reviewed in Hardie and Juusola, 2015). Arrestin 1 (ARR1) and Arrestin 2 (ARR2) function to turn off the signal by interacting with Rhodopsin (Dolph et al., 1993). When Rhodopsin is activated by light, ARR2 is recruited to the rhabdomere within 5 min of light exposure to interact with Rhodopsin and deactivate it (Figure 1, visual adaptation; Ranganathan and Stevens, 1995). Due to deactivation of the signal by Arrestins, more Rhodopsins will be in the inactive state in light than in dark, thereby downregulating the visual sensitivity under high light intensity (Song and Juusola, 2017).

Other light-dependent changes that can down regulate visual sensitivity occur more slowly. These include changes in the INAD complex and movement of TRPL (Bahner et al., 2002) and Gα proteins (Kosloff et al., 2003; Cronin et al., 2004) out of the rhabdomere, so that they will no longer respond to the rhabdomeric signals from Rhodopsins (Figure 1, light adaptation). In the dark, most TRPL channels and Gα proteins are in the rhabdomeres, while in the light their subcellular localization has changed to the cell body outside of the rhabdomere. TRPL is a transmembrane channel, and its localization continues to be in the cell membrane (Cronin et al., 2006). These changes are thought to be produced by the Rhodopsin signaling pathway (Meyer et al., 2006) but then produce less stimulation of the visual transduction pathway by light under high illumination (Bahner et al., 2002), so that the downstream synaptic signaling is not saturated and can still respond to changes in light intensity.

## The circadian molecular oscillation in Drosophila

The circadian oscillation is generated intracellularly by oscillation in the level and location of the Period (PER) protein. The genetic analysis of circadian rhythms in flies has generated a molecular circadian clock model that is considered a triumph for modern molecular genetics and recently earned a Nobel Prize in Physiology or Medicine for Michael Rosbash, Jeffrey Hall and Michael Young (Siwicki et al., 2018). The model posits interlocked transcriptional and post-translational feedback loops (Figure 1, circadian rhythm). Drosophila circadian repressors like PER accumulate to repress transcriptional activation by the transcription factor complex Clock/Cycle (CLK/CYC), while accumulation of TIM and PER is antagonized, respectively, by light-dependent degradation mediated by the CRY photoreceptor and proteolysis triggered by kinases like Double time (DBT) (Siwicki et al., 2018). The activities of CRY and DBT establish a post-translational feedback loop that is essential to convert the negative transcriptional feedback processes regulating *per* and *tim* transcription into a molecular oscillator. The DBT kinase binds to PER and phosphorylates it slowly in the cytosol to trigger its degradation and prevent its nuclear accumulation, and therefore *per* and *tim* mRNA can rise to a peak during the day, uninhibited by the transcriptional negative feedback that PER would otherwise exert on these promoters (Kloss et al., 1998; Price et al., 1998). When lights go out in the evening, TIM protein can accumulate because its degradation is no longer produced by the CRY photoreceptor (Emery et al., 1998; Stanewsky et al., 1998). TIM is capable of antagonizing PER degradation (Price et al., 1995) and mediates PER translocation to the nucleus (Vosshall et al., 1994; Jang et al., 2015), where PER represses *per* and *tim* transcription until the early day, when light again triggers TIM degradation, and PER phosphorylation by DBT eventually triggers degradation of PER, thereby relieving the transcriptional repression of *per* and *tim*. In this manner, out of phase oscillations of PER/TIM protein and mRNA are generated, rather than a constitutively expressed homeostasis that a negative feedback loop would typically produce (Kloss et al., 1998; Price et al., 1998). The cycles of PER-dependent transcription drive rhythmic transcription of many other genes that are clock outputs; these do not contribute to the oscillation of PER and PER-dependent transcription but do provide numerous daily changes (e.g., in metabolism and sleep–wake activity) that generate the multiple organismal manifestations of circadian rhythms.

The Drosophila circadian clock in the eye is thought to be sustained by the same oscillations of PER protein that drive the circadian clock of the brain. Both the eye and the brain exhibit circadianly controlled PER oscillations (Zerr et al., 1990). PER oscillations are detected in dissected eyes; in fact 90% of the PER oscillation detected by immunoblot analysis of heads comes from the eye and not the brain, although oscillations of PER occur in both the brain and the eye (Zeng et al., 1994). Moreover, the intracellular mechanisms of the eye and brain clocks operate independently because the oscillations of *per* mRNA can be suppressed by overexpressing PER protein in the eyes with no effects on *per* mRNA in other cells or on locomotor activity, which is controlled by the brain cells (Zeng et al., 1994). Mutants in the CRY photoreceptor eliminate entrainment of the eye circadian PER oscillations to light, even though entrainment of the central brain PDF^+^ neurons that control locomotor activity continue in *cry* mutants because of the neural signals from the eye to the brain neurons (Stanewsky et al., 1998). Finally, we have shown the expression of *bdbt* RNAi in the eye dampens the circadian oscillations of PER and DBT in the eye with no effects on circadian behavior (Nolan et al., 2023), while expression of *bdbt* RNAi more broadly in brain circadian cells, which also express BDBT, has strong effects on behavior (Fan et al., 2013). It is widely accepted that the photoreceptors have one of several independent circadian clocks in fly but the functions of the eye clock are not known. Could one manifestation of the circadian clock in the eye be to control the timing of visual sensitivity changes?

## BDBT is a protein whose association with DBT is necessary for circadian rhythms, and it is regulated by both light and circadian rhythms in the eye

Bride of DBT (BDBT) was discovered by our lab in a proteomic screen for factors that associate with the principal circadian kinase DBT (hence the name), and it is required for normal DBT activity on PER protein (stimulation of DBT activity, Figure 1; Fan et al., 2013). Knock down of BDBT with RNAi in all circadian cells produced long periods and arrhythmicity, with reductions in the oscillations of PER level, nuclear localization and phosphorylation state in the head. These results were consistent with a stimulation of DBT activity towards PER by BDBT (Fan et al., 2013). Our collaboration derived an X ray crystallography analysis of BDBT’s structure, thereby showing that it is an FK506 binding protein (Fan et al., 2013). However, it did not have the structure or residues to bind FK506 or isomerize the cis-trans conformations at prolines, so it is a “noncanonical” FK506 binding protein. These are typically involved in assembling macromolecular complexes.

Our antibody to BDBT generated a dramatic change in its immunofluorescence during the day, but only in the eye and not in other parts of the brain where it is expressed constantly (Fan et al., 2013). During the night, it accumulated into a broad but punctate expression pattern (foci) in all photoreceptors. During the day, it largely disappears from view except in photoreceptor cell #7, which expresses UV-sensitive rhodopsins 3&4, generating fingers of expression on the outside of the eye (Nolan et al., 2023). In *per^o^* and *dbt* RNAi expressing flies, the broadly expressed foci never accumulate in the dark, demonstrating their requirement for a functional circadian clock (Fan et al., 2013). In addition, in response to constant light and constant darkness BDBT is expressed in fingers and broadly, respectively (Nolan et al., 2023).

Our analysis of visual and circadian transduction mutants showed a requirement for both circadian and visual photoreceptors, suggesting involvement of BDBT in both the circadian and visual pathways. Mutants in the *cry* photoreceptor as well as the Rhodopsin 1 photoreceptor (*ninaE,* expressed in 6 of the eye photoreceptors) produced constitutively broad levels of BDBT foci in both light and dark (inhibition of BDBT foci by both CRY and Rh1 in Figure 1 and Nolan et al., 2023). *NinaE* mutants do not affect PER oscillations in the eye (Zerr et al., 1990) while *cry* mutants eliminate PER oscillations in the eye (Stanewsky et al., 1998), demonstrating possibly a unique requirement for both circadian and visual transduction for BDBT oscillations. Moreover, mutations in Arrestin 1 (*Arr1*) and Arrestin 2 (*Arr2*) both produce an absence of broad BDBT expression in both the light and the dark (Nolan et al., 2023) – the opposite phenotype from the *ninaE* mutations. This is consistent with the antagonism of Rhodopsin signaling by Arrestins; in their absence Rhodopsin signals would be elevated and may suppress BDBT even in the dark (inhibition of Rhodopsin by Arrestins, Figure 1).

Intriguingly, the daily and mutant changes in BDBT foci are not produced by changes in BDBT levels, as immunoblot analysis of isolated eyes shows constant levels of expression throughout the day and equivalent levels of expression in wild type and mutant flies. Elevating the temperature of the immunofluorescence detection to 60°C after paraformaldehyde fixation and before antibody detection elevated the BDBT signal in the eyes of light-adapted flies to the same level as those from dark adapted flies (Nolan et al., 2023). Evidently, BDBT moves into complexes in which its epitopes are masked during the day.

It is likely that this change in antigenicity involves changes in the rhabdomeric structure. After only 5 min of light exposure, the rhabdomeric contractions noted above (Hardie and Franze, 2012) lead to loss of immunofluorescent signal for Rhodopsins, TRP and TRPL channels (Schopf et al., 2019). Recent work by the Huber lab has shown that the loss is due to epitope masking, because GFP-tagged or SNAP-tagged versions of the TRPL channels are still detected in the rhabdomeres by their intrinsic fluorescent signals immediately after light exposure but not by antibodies (Schopf et al., 2019). The TRPL channel moves out of the rhabdomere, so its masking is only temporary because it can be visualized again once it is in the cell body. Could BDBT reside in the rhabdomeres during the entire light phase and be permanently masked during the light phase by the altered state of the rhabdomeres? What function (if any) does it have in the rhabdomere?

## Discussion

### Could BDBT, CRY and the circadian clock be involved in mediating changes in visual sensitivity in response to light intensity changes?

As noted above, Gα and TRPL move out of the rhabdomere in response to light to down regulate the visual sensitivity to light. Gα and TRPL also move into of the rhabdomere in response to darkness (Bahner et al., 2002; Kosloff et al., 2003; Cronin et al., 2004). Could BDBT be involved in signaling these movements, perhaps via interactions with the circadian clock and the rhabdomere (Figure 1, “1?”)? It could move into the rhabdomere in response to light to regulate these movements, with suppression of its detection during the light-mediated contraction of the rhabdomere. Clear detection of BDBT in the light phase will involve construction of transgenic flies in which BDBT is tagged with an intrinsically fluorescent tag like GFP or SNAP to avoid masking of antibody access. It may interact with some of these visual factors to modify their locations in the eye or to allow access to them by other components of the circadian system like DBT. Intriguingly, other work has shown a light-inducible form of DBT that is regulated by LARK-mediated translational control of an alternative transcript (Huang et al., 2014).

Like BDBT, the CRY photoreceptor may be involved in both visual and circadian processes. The involvement of CRY in the regulation of BDBT in the eye suggests the circadian light detection may be involved in regulation of BDBT foci and possibly Gα and TRPL movements, because CRY interacts with TIM in response to light to trigger degradation of TIM (Emery et al., 1998; Stanewsky et al., 1998; Ceriani et al., 1999; Busza et al., 2004; Koh et al., 2006), thereby resetting the phase of the clock (inhibition of TIM, Figure 1). However, CRY also interacts with the visual transduction apparatus to confer light-mediated regulation of the pathway (stimulation of Rhodopsin, Figure 1; Mazzotta et al., 2013; Schlichting et al., 2018). The C terminus of CRY is involved in interactions with the rhabdomeric visual signaling complexes via its interactions with PDZ domains in INAD during the light (Mazzotta et al., 2013). *cry^O^* and *cry^M^* mutants missing this domain do not show daily changes in their visual responses that wild type flies show, indicating a role for the most C terminal PDZ-binding domain in CRY’s light-dependent signaling to the visual system. Another effect of CRY on visual transduction was shown by the finding that CRY interacts with actins in a light independent manner and thereby localizes to the rhabdomere in both light and dark; this localization enhanced the sensitivity of the eye to light and does not require CRY’s direct response to light because it is also observed with red light to which CRY’s do not respond (Schlichting et al., 2018). Therefore, like BDBT, CRY is another circadian component that could regulate visual adaptation to light (Figure 1, “1?”).

If the circadian oscillator in general is involved in regulation of visual sensitivity, knock-down of BDBT in the eye and other circadian mutants (eg, *dbtRNAi, per^o^, per^s^* and *per^l^*) should alter the cycles of TRPL and Gα into and out of the eye (Figure 1, “2?”). As noted above, the *per^o^* and *dbt* RNAi genotypes eliminate the formation of BDBT foci in the dark. It seems more likely that the circadian clock would regulate the relatively slow time course of TRPL and Gα movements (Changes in TRPL subcellular localization are detected from 2 to 16 h after light exposure; Schopf et al., 2019) than for the relatively rapid responses of Ca^++^-triggered and Arrestin-dependent down-regulation of Rhodopsin signaling, which clearly responds immediately to light. Nevertheless, the circadian clock might also regulate the sensitivity of these other rapid responses to light intensity. Do TRPL and Gα exhibit persistent circadian oscillations of location in constant darkness? It is also possible that the brain circadian clock could control the visual sensitivity of the eye, as vision also involves the response of the brain to photoreception.

### The relevance of these findings to human eyes

The signaling pathway downstream of vertebrate photoreceptor retinal Rhodopsins differs greatly from that of Drosophila Rhodopsins and photoreception, as the second messenger is cGMP, which is reduced in response to light, and therefore light produces closing of cGMP-gated channels (reviewed in Hardie and Juusola, 2015). On the other hand, the signaling pathway found in Drosophila photoreceptors is found in intrinsically photosensitive retinal ganglion cells, which entrain circadian rhythms in the brain SCN and control pupillary diameter (Provencio et al., 1998; Hattar et al., 2003; Panda et al., 2005; Qiu et al., 2005; Xue et al., 2011). The alterations of visual sensitivity to dim and bright light are mediated by two different types of human photoreceptor cells (rods and cones), and the role of Ca^++^ (Vinberg et al., 2018) and light-dependent movement of G proteins and Arrestins from signaling complexes in response to light are common features with the Drosophila photoreceptors, despite the differences in downstream signals emanating from light. These changes in location and interactions thereby reduce the signals that are produced by stimulation of Rhodopsin.

The circadian clock is known to be involved in the regulation of mammalian visual sensitivity. Circadian oscillations of clock proteins are observed in photoreceptors of the mammalian eye (Liu et al., 2012) and knock out of the circadian clock only in the retina produces a loss of the circadian oscillation in the ERG (Storch et al., 2007; Sengupta et al., 2011), suggesting that a retinal clock regulates the circadian control of the visual response. Disruption of circadian rhythms in the eyes of mammals causes deficits in the eye as well, demonstrating the importance of the rhythms for normal functioning of the eye (reviewed in DeVera et al., 2019). While FK506 binding proteins have been shown to interact with CKI epsilon and delta (the two mammalian orthologs of DBT; Kategaya et al., 2012), there is no clear ortholog of BDBT in mammals; clear BDBT orthologs are only found in flies (diptera) and one locust (Thakkar et al., 2022). Moreover, the nuclear localization signal to which BDBT binds (Venkatesan et al., 2015, 2019) is preceded by an Asparagine (N) in Drosophila and by a phosphorylatable Threonine (T) in mammals and yeast (Cullati et al., 2022; Thakkar et al., 2022). This T can be phosphorylated in mammals and yeast to change the substrate preference for CKI. Since Drosophila DBT is missing this T and has BDBT, it is not clear at the moment whether the mammalian FKBPs would serve a similar role in the mammalian circadian rhythm or regulation of visual sensitivity. In any event, the possible evolutionary divergence or similarity of visual sensitivity mechanisms is an interesting topic for future research.

## Data availability statement

The raw data supporting the conclusions of this article will be made available by the authors, without undue reservation.

## Ethics statement

The manuscript presents research on animals that do not require ethical approval for their study.

## Author contributions

RN: Conceptualization, Writing – review & editing. J-YF: Conceptualization, Writing – review & editing. JP: Conceptualization, Funding acquisition, Writing – original draft, Writing – review & editing.
